# Altered Degree Centrality of Brain Networks in Parkinson's Disease With Freezing of Gait: A Resting-State Functional MRI Study

**DOI:** 10.3389/fneur.2021.743135

**Published:** 2021-10-11

**Authors:** Chaoyang Jin, Shouliang Qi, Yueyang Teng, Chen Li, Yudong Yao, Xiuhang Ruan, Xinhua Wei

**Affiliations:** ^1^College of Medicine and Biological Information Engineering, Northeastern University, Shenyang, China; ^2^Key Laboratory of Intelligent Computing in Medical Image, Ministry of Education, Northeastern University, Shenyang, China; ^3^Department of Electrical and Computer Engineering, Stevens Institute of Technology, Hoboken, NJ, United States; ^4^Department of Radiology, Guangzhou First People's Hospital, School of Medicine, South China University of Technology, Guangzhou, China

**Keywords:** Parkinson's disease, freezing of gait, resting-state fMRI, functional brain network, degree centrality

## Abstract

Freezing of gait (FOG) in Parkinson's disease (PD) leads to devastating consequences; however, little is known about its functional brain network. We explored the differences in degree centrality (DC) of functional networks among PD with FOG (PD FOG+), PD without FOG (PD FOG–), and healthy control (HC) groups. In all, 24 PD FOG+, 37 PD FOG–, and 22 HCs were recruited and their resting-state functional magnetic imaging images were acquired. The whole brain network was analyzed using graph theory analysis. DC was compared among groups using the two-sample *t*-test. The DC values of disrupted brain regions were correlated with the FOG Questionnaire (FOGQ) scores. Receiver operating characteristic curve analysis was performed. We found significant differences in DC among groups. Compared with HCs, PD FOG+ patients showed decreased DC in the middle frontal gyrus (MFG), superior temporal gyrus (STG), parahippocampal gyrus (PhG), inferior temporal gyrus (ITG), and middle temporal gyrus (MTG). Compared with HC, PD FOG– presented with decreased DC in the MFG, STG, PhG, and ITG. Compared with PD FOG–, PD FOG+ showed decreased DC in the MFG and ITG. A negative correlation existed between the DC of ITG and FOGQ scores; the DC in ITG could distinguish PD FOG+ from PD FOG– and HC. The calculated AUCs were 81.3, 89.5, and 77.7% for PD FOG+ vs. HC, PD FOG– vs. HC, and PD FOG+ vs. PD FOG–, respectively. In conclusion, decreased DC of ITG in PD FOG+ patients compared to PD FOG– patients and HCs may be a unique feature for PD FOG+ and can likely distinguish PD FOG+ from PD FOG– and HC groups.

## Introduction

Freezing of gait (FOG) is a disabling condition that often affects people with advanced-stage Parkinson's disease (PD) (1). PD with FOG (PD FOG+) patients have difficulty in walking effectively (2, 3) and their quality of life and overall health are also greatly affected (4). The clinical manifestation of FOG is that when the patients attempt to walk or advance, their steps are suddenly interrupted or significantly reduced. PD FOG+ patients often report that their feet seem stuck to the floor or sucked by the floor, making it difficult to lift their feet and/or step forward (5). The typical FOG symptom usually lasts for a few seconds, but occasionally can last for 30 s or more (6). Unlike other cardinal symptoms, FOG cannot be satisfactorily managed by dopaminergic medication or deep brain stimulation (7, 8).

At present, there is no unified understanding of the pathophysiological mechanism of FOG (9, 10). Some recent studies suggest that PD FOG+ patients have difficulty in performing movements, which leads to them focusing more on the execution of the said movement (11, 12), while other studies indicate that PD FOG+ patients have executive function disorder and cannot perform well when they are required to respond (4, 13). In addition, FOG also includes several important aspects such as impaired motor rhythm control, loss of bilateral coordination, and asymmetry of gait [(14–16)]. In recent years, an increasing number of neuroimaging studies have shown alternations in brain structure and function in PD FOG+ patients. Structural MRI studies have shown gray matter loss and white matter damage in different cortical and subcortical areas including the frontal and parietal cortex, brainstem, and basal ganglia in PD FOG+ patients (17, 18). Novel insights indicate that FOG is related not only to specific brain structure damage but also to brain functional alterations.

Resting-state functional magnetic resonance imaging (rs-fMRI) reflects neuronal activity in the resting state and can be used to detect functional connectivity (FC) and large-scale brain network organization *via* blood oxygen level dependent (BOLD) signal fluctuation. Brain regions of cortico-striatal network have been found of the abnormal neural oscillations in the in the slow 4- and slow 5-band in PD, suggesting a frequency-dependent activity (19). Between putamen and supplementary motor regions, compared with HC, PD patients present the enhanced functional connectivity (20). PD pathology triggers the regions of executive control network less than the those in default mode network (21).

Wang et al. reported that PD FOG+ patients showed abnormal pedunculopontine nucleus FC, which was mainly concentrated in the corticopontine-cerebellar pathways along with the visual temporal areas (22). Using the method of independent component analysis (ICA), researchers have found that the functional connection of executive attention and visual neural network is interrupted in PD FOG+ patients (23, 24). Current studies have shown that extensive functional damage of cortical and subcortical brain structures, involving multiple brain networks, is closely related to FOG in PD. However, these studies did not integrate the specificity of brain regions and connectivity between different brain regions into an analytical framework for patients with FOG. From the perspective of brain function network, studies are ongoing with respect to further investigations regarding the mechanism of FOG. Most of these previous studies have mainly focused on the functional connections (FC) in specific brain sub-networks; however, the topological organization of the whole brain functional network in FOG is still poorly understood.

Graph theory has become an increasingly powerful tool to study the topological characteristics of brain networks, and complex brain network analysis technology based on graph theory is an area of focus in current neuroscience research (25–29). Typically, to complete a simple task, the human brain relies on the interaction and coordination of various functional areas. Studies on the human brain system from the perspective of complex networks, especially the topological structure formed by the interaction of various functional areas, may aid researchers to further investigate the mechanism of information processing and mental expression in the brain (30, 31). Some studies have reported changes in the connectivity within the sensorimotor network and the interaction between the visual network and other brain modules in Parkinson's patients (32–34). In addition, significant differences of topological characteristics between PD and HC are reported in the default mode network and the occipital region (35, 36).

Research on the topological properties of resting functional brain networks is helpful to understand the pathophysiology of diseases (37). Degree centrality (DC), as a graph theory measure, is the most direct indicator of node centrality in brain network analysis (38). DC reflects the importance of nodes in the entire network and their information communication capabilities. The greater the DC of a node, the more important the node is in the entire brain network, and the stronger its information communication capabilities (39). Therefore, DC is a very important topological property in the functional brain network.

In this study, we explored the abnormalities of DC in the functional brain network among three groups, namely, PD FOG+, PD FOG–, and healthy controls (HCs). For this, the GRaph thEoreTical Network Analysis (GRETNA) toolbox was used to construct the entire functional brain network, and graph theory analysis (GTA) method was used to analyze the abnormalities of DC attributes. The DC values of disrupted brain regions in PD FOG+ were correlated to the FOG Questionnaire (FOGQ) score. Receiver operating characteristic (ROC) curve analysis was performed to evaluate the discriminative capability of related DC among the three groups.

## Materials and Methods

### Participants

In this study, we enlisted 61 right-handed PD patients. All patients met the diagnostic criteria of the UK Brain Bank for PD. Patients were excluded if the following conditions were met: (1) Severe tremor, severe brain injury, musculoskeletal disease, history of stroke; (2) Serious impact on gait stability: visual impairment diseases, bone and joint diseases, musculoskeletal diseases; (3) Significant cognitive dysfunction [Mini-Mental State Examination (MMSE) score <24]; and (4) Unsuitable for MRI because of claustrophobia or having metal implants. The criteria for patients deemed to have PD FOG+ were as follows: (1) The patients' FOGQ scores were >1; and (2) After a series of exercise tests, two or more experienced neurologists determined that patients have FOG.

We recruited 22 right-handed healthy controls from the community through poster advertisements, including 13 female and 9 male. The exclusive criteria of HCs are given as follows: (1) MMSE score is <24; (2) Any (other) major systemic, mental or neurological diseases (i.e., depression, dementia) is available; (3) Focal or diffuse brain injury as determined by conventional MRI, including defects and extensive cerebrovascular diseases, is available.

Finally, 83 participants are included in this study, including 24 PD FOG+ patients, 37 PD FOG– patients, and 22 healthy controls matched by age, sex, and education level. This study was approved by the Ethics Committee of Guangzhou First People's Hospital and was conducted in adherence with the 1964 Declaration of Helsinki and its subsequent amendments or similar ethical standards. All subjects signed an informed consent form before participating in this study.

### Clinical Assessment

We evaluated all PD patients with respect to motor and cognitive abilities, including general cognition and executive functions in particular. The Unified Parkinson's Disease Rating Scale (UPDRS-III) (40) and the Hoehn and Yahr (H&Y) scale were used to assess the severity of motor symptoms (41). The Timed Up and Go (TUG) test was used to evaluate patients' functional walking ability (42). The FOGQ score was employed to evaluate the severity of FOG. Both MMSE and Montreal Cognitive Assessment (MoCA) were used to assess intellectual status and cognitive function. The Frontal Assessment Battery (FAB) (43) test was used to assess executive functions related to the frontal lobes. Both Hamilton Anxiety Rating Scale (HARS) and Hamilton Depression Rating Scale (HDRS) were used to assess depression and anxiety levels.

### MRI Data Acquisition

A 3.0T MAGNETOM Verio whole body MRI system (Siemens, Munich, Germany) equipped with an eight-channel phased-array head coil was used for MRI scanning. To eliminate the effect of drugs on neural activity, all PD patients were in the “off” state for image acquisition (i.e., patients were asked to stop taking anti-Parkinson's drugs for at least 12 h). Further, to reduce the subjects' head movement and noise as much as possible, tight foam padding and noise-reducing earplugs were used. All participants were guided to keep still, stay awake, close their eyes, and avoid thinking about anything as much as possible.

Three-dimensional (3D) anatomical images were acquired with T1-weighted sequence. The repetition time (TR) was 1,900 ms; echo time (TE), 102 ms; flip angle, 9°; thickness, 1.0 mm; number of slices, 160; field of view (FOV), 250 × 250 mm^2^; dimension of matrix, 256 × 256; and voxel size, 1.0 × 1.0 × 1.0 mm^3^. The rs-fMRI images were obtained by using echo-planar imaging (EPI) sequence (TR was 2,000 ms; TE, 21 ms; flip angle, 78°; FOV, 192 × 192 mm^2^; dimension of matrix, 64 × 64; time points, 220; slice thickness, 3.0 mm; and voxel size, 3 × 3 × 3 mm^3^). The flip angle of 78° is based on the comprehensive consideration of the signal-to-noise ratio and contrast of the scanned image. The larger the flip angle, the higher the signal-to-noise ratio of the image, but the contrast of the image will decrease. Considering the tradeoff of the signal-to-noise ratio and contrast, we finally chose a flip angle of 78°.

### Overview of the Study Procedure

**Figure 1:**
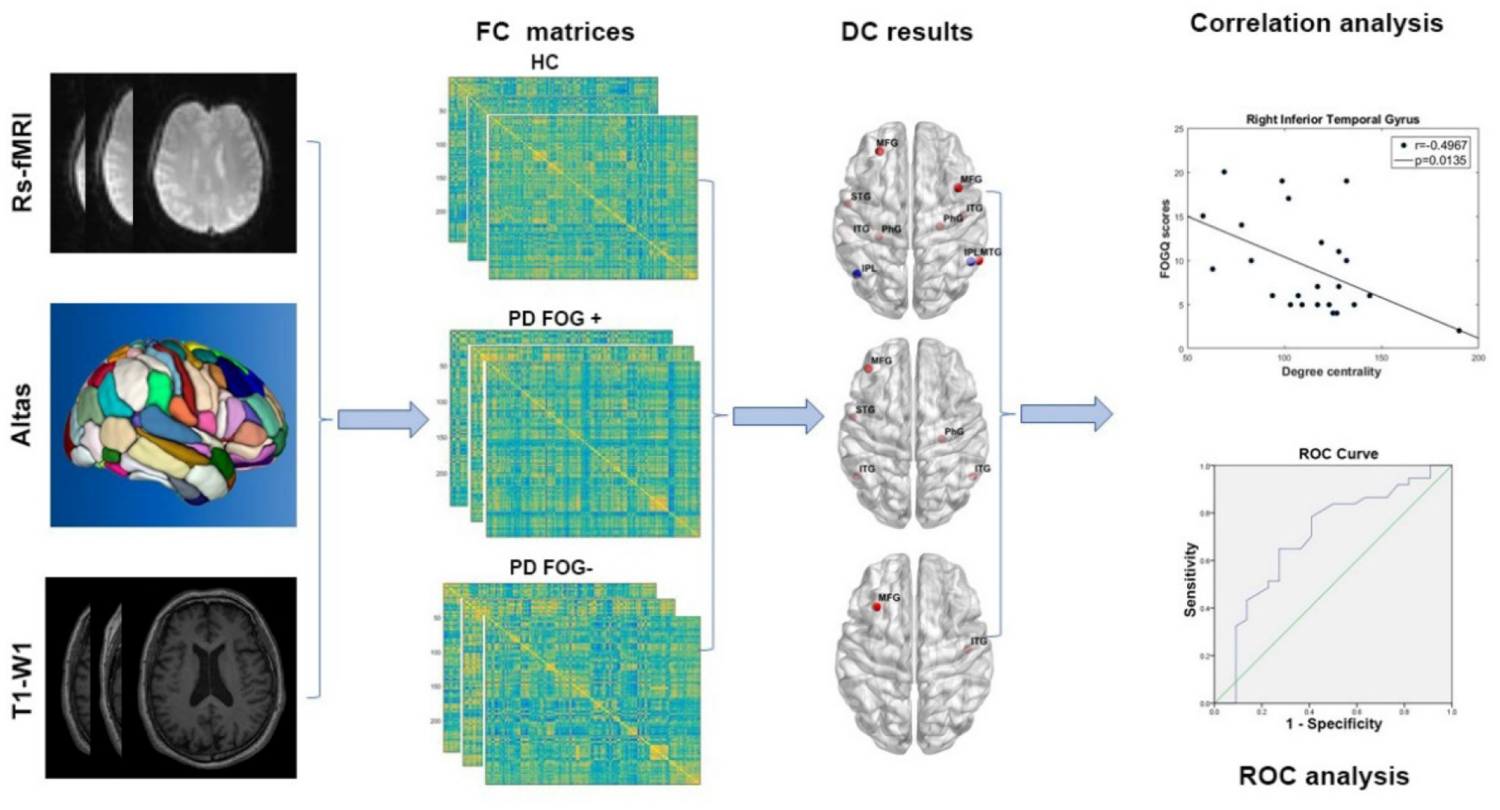
The study procedure of image preprocessing, functional network construction, DC, correlation, and ROC analysis (rs-fMRI, resting-state functional magnetic resonance imaging; T1-WI, T1-weighted imaging; FC, functional connectivity; HC, healthy control; PD FOG+, Parkinson's disease with freezing of gait; PD FOG−, Parkinson's disease without freezing of gait; DC, degree centrality; ROC, receiver operating characteristic curve).

There were five main steps in our study procedure (Figure 1): (1) Image preprocessing was conducted for rs-fMRI and T1-weighted images. (2) Functional brain network was constructed and FC matrices were calculated. (3) Two-sample *t*-test was used to identify significant differences in DC in all brain regions across the three groups. (4) Correlation analysis was performed between the DC value of disrupted brain regions in the PD FOG+ group and FOGQ score. (5) ROC curve analysis was carried out to evaluate the discriminative capability of related DC among the three groups.

### Preprocessing of fMRI Data

Data Processing Assistant for Resting-State fMRI (DPARSF) edition V5.4 was applied to preprocess the rs-fMRI data (44). Briefly, the preprocessing steps include the following: (1) image conversion from DICOM format to NIFIT format; (2) removing the first 10 time points of each subject's image to eliminate non-equilibrium effects of magnetization and allowing subjects to get used to the scanning environment; (3) time correction between slices to eliminate the difference of image acquisition; (4) T1-weighted images were co-registered to EPI scans; (5) the method of “New Segment + Dartel” was used for gray matter, cerebrospinal fluid (CSF), and white matter (WM) segmentation; (6) spatial normalization with resampling to a voxel size of 3 × 3 × 3 mm^3^; (7) spatial smoothing (Gaussian kernel with 6-mm full width at half maximum); (8) temporal bandpass filtering at low frequency (0.01–0.08 Hz); and (9) nuisance regression (including WM signal, CSF signal, and six head motion parameters).

Besides the visual inspection, we have the measures to evaluate the registration of T1 and EPI scans for all subjects. If the head motion in the registration is larger than 2 mm (translation) or 2° (angular rotation), this subject will be excluded for the further study. The related contents have been added into the revised manuscript.

### Network Construction

The construction of functional brain network was based on the GRETNA toolbox (https://www.nitrc.org/projects/gretna) (45). Previous studies on brain network construction and computation also applied this method (46–49). Using the Human Brainnetome Atlas, the whole brain was divided into 48 brain regions which are further divided into 246 subregions (no. 1–246) (50). Each subregion represents a network node. The Pearson correlation coefficients were used for calculating the edges of brain network. Finally, the symmetric functional connectivity matrix with 246 × 246 was constructed for further network analysis.

### Network Metrics

The GTA method was applied to calculate DC, which is one of most important topological properties of functional brain network at the regional level. DC is the most direct measure to describe the centrality of nodes in network analysis. The larger the DC of a node, the more important the node is in the network. In addition, it acts as the hub of network information transmission in the functional brain network (38).

The area under the curve (AUC) of each matrix was calculated by using the GRETNA toolbox (45). The AUC could provide an outline scalar quality for topological properties of functional brain network, which is independent of signal threshold value selection. In addition, it is also very sensitive to topological changes in neurological diseases (46, 51). The matrix of each subject will be set with a threshold, which will produce a binary undirected network. The network has different link densities, ranging from 0 to 100% in increments of 1%.

### Statistical Analysis

Demographic and clinical characteristics of all subjects in different groups were compared and analyzed through Statistical Product and Service Solutions (SPSS), version 24.0 (52). ANOVA with *post-hoc* tests is utilized for comparing variables between the three groups (HC, PD FOG+, and PD FOG–). The Mann–Whitney *U*-test is for comparing variables between two groups. The gender of participants is compared by using the chi-squared test.

For each brain region, DC is compared for PD FOG+ vs. HCs, PD FOG– vs. HCs, and PD FOG+ vs. PD FOG–. Specifically, in the DC comparison, the age, gender, and disease duration have been taken into account as the covariates by using a pairwise two-sample *t*-test. False discovery rate (FDR) was used to perform multiple comparison corrections. *p* < 0.05 indicated a significant difference.

In both PD FOG+ and PD FOG– groups, the brain regions that showed significant difference (*p* < 0.05) were selected as ROI. Then, the DC values of these abnormal brain regions were extracted and correlated with the FOGQ scores of PD FOG+ patients to obtain the Pearson correlation coefficient. In addition, the extracted DC values were also used for ROC analysis (53). In the ROC analysis, we reported the sensitivity, specificity, correct classification, and AUC for the cut-off value (54). The choice of cut-off value was to use the Youden's index and the shortest distance from the coordinate (0, 1) on the ROC curve. The closer the AUC value was to 1, the stronger the classification ability of the findings.

## Results

### Demographic and Clinical Characteristics

Demographic and clinical characteristics of the participants are summarized in Table 1. As for age, education level, sex, and MMSE scores, no significant differences have been found between HC, PD FOG–, and PD FOG+ groups (*p* > 0.05). PD FOG+ patients showed significant differences in disease duration, FOGQ, FAB, and TUG scores (*p* < 0.05), but there was no difference in terms of UPDRS-III score, H&Y scale score, MoCA, HDRS, or HARS scores (*p* > 0.05) between PD FOG+ and PD FOG– groups.

**Table 1:** Demographic and clinical characteristics of participants.

**Parameter**	**HC**	**PD FOG+**	**PD FOG–**	***p*** **-value**
	**(*n* = 22)**	**(*n* = 24)**	**(*n* = 37)**	
Age, years	62.5 ± 3.8	65.5 ± 6.1	64.1 ± 8.2	0.298^a^
Education, years	10.98 ± 2.34	9.35 ± 3.42	10.65 ± 4.25	0.36^a^
Sex, female/male	13/9	11/13	18/19	0.452^b^
Disease duration, years	NA	6.00 ± 5.25	3.01 ± 3.21	0.0083^c^
UPDRS-III	NA	22.4 ± 6.72	21.34 ± 10.30	0.18^c^
H and Y scale	NA	2.49 ± 0.51	2.07 ± 0.49	0.17^c^
FOGQ	NA	9.25 ± 5.87	1.54 ± 1.67	<0.001^c^^*^
MMSE	26.58 ± 1.62	25.27 ± 4.01	25.71 ± 4.25	0.582^a^
MoCA	NA	21.08 ± 4.77	21.89 ± 5.66	0.372^c^
FAB	NA	13.8 ± 2.6	15.7 ± 1.5	<0.001^c^^*^
TUG	NA	12.5 ± 1.6	1.9 ± 0.7	<0.001^c^^*^
HDRS	NA	7.82 ± 6.35	9.67 ± 6.23	0.37^c^
HARS	NA	11.32 ± 6.74	10.56 ± 7.56	0.69^c^

*Data are shown as mean ± standard deviation*.

* *p < 0.05;*

a *one-way analysis of variance;*

b *chi-squared test;*

c *Mann–Whitney U-test*.

*FAB, Frontal Assessment Battery; FOGQ, Freezing of Gait Questionnaire; H and Y, Hoehn and Yahr; HARS, Hamilton Anxiety Rating Scale; HDRS, Hamilton Depression Rating Scale; HC, healthy control; MMSE, Mini-mental State Examination; MoCA, Montreal Cognitive Assessment; NA, not applicable; PD FOG+, Parkinson's disease with freezing of gait; PD FOG–, Parkinson's disease without freezing of gait; TUG, Timed Up and Go; UPDRS, Unified Parkinson's Disease Rating Scale*.

### Difference in DC Among Groups

**Figure 2:**
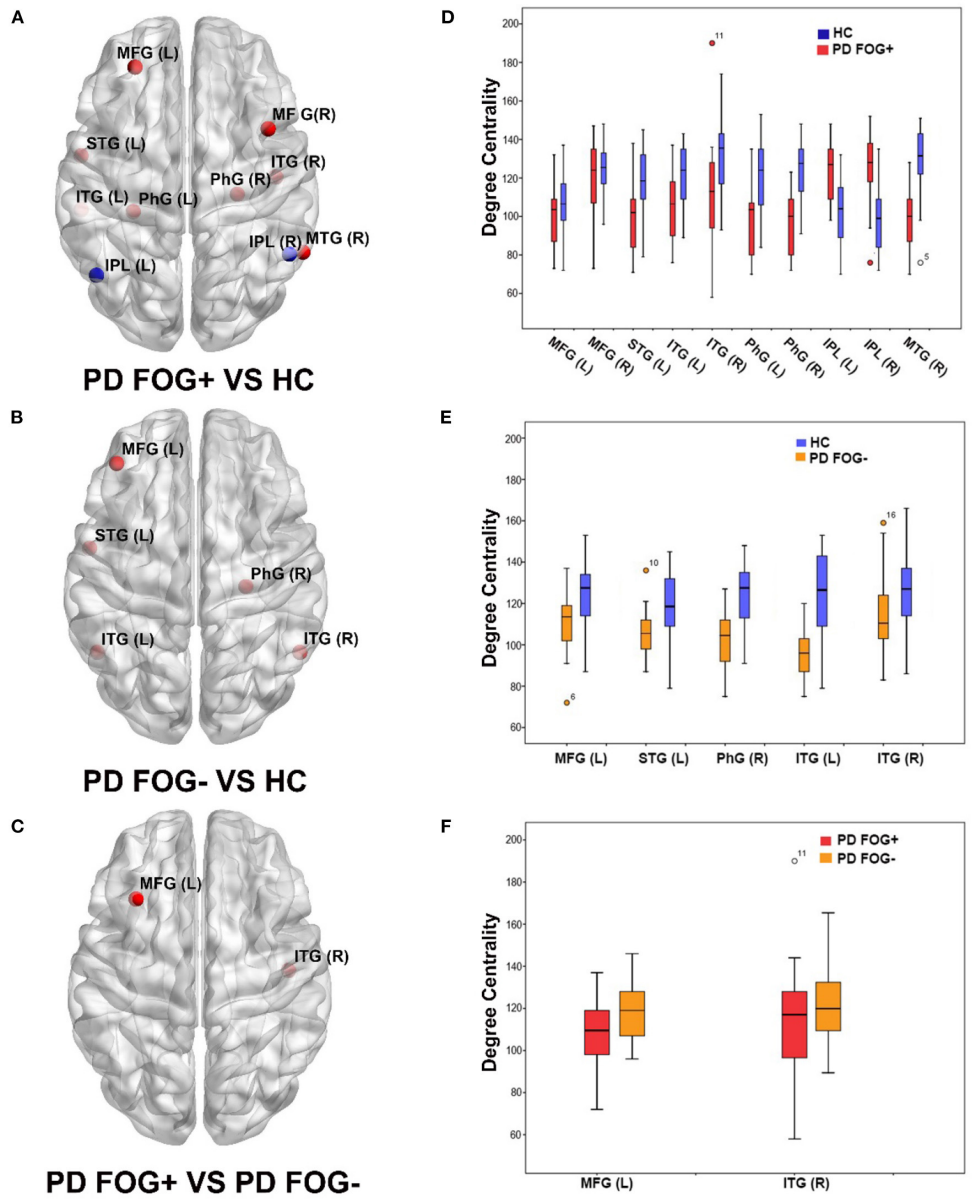
Brain regions showed significant differences in degree centrality (DC) among three groups (*p* < 0.05, FDR correction). **(A)** Brain regions with significant DC in PD FOG+ vs. HC. **(B)** Brain regions with significant DC in PD FOG– vs. HC. **(C)** Brain regions with significant DC in PD FOG+ vs. PD FOG–. **(D)** DC values of brain regions showing significant differences in PD FOG+ vs. HC. **(E)** DC values of brain regions showing significant differences in PD FOG– vs. HC. (**F**) DC values of brain regions showing significant differences in PD FOG+ vs. PD FOG–.

As shown in Figure 2, significant difference in the nodal DC was observed in some brain regions among the PD FOG+, PD FOG–, and HC groups. Compared with HCs, PD FOG+ patients showed decreased DC in the left and right middle frontal gyrus (MFG), left superior temporal gyrus (STG), left and right parahippocampal gyrus (PhG), left and right inferior temporal gyrus (ITG), and right middle temporal gyrus (MTG) and increased DC in the left and right inferior parietal lobule (IPL) (Figures 2A,D). Compared with HCs, PD FOG– patients showed decreased DC in the left MFG, left STG, right PhG, and left and right ITG regions (Figures 2B,E). Compared to PD FOG– patients, PD FOG+ patients showed decreased DC in the right ITG and left MFG (Figures 2C,F).

In the comparison of PD FOG+ vs. HCs and PD FOG– vs. HCs, there were five common regions with decreased DC, comprising left MFG, left STG, right PhG, and left and right ITG. More regions including the right MFG, right MTG, and left PhG with decreased DC were observed in PD FOG+ vs. HCs than in PD FOG– vs. HCs.

### Correlation Between DC and FOG Severity

**Figure 3:**
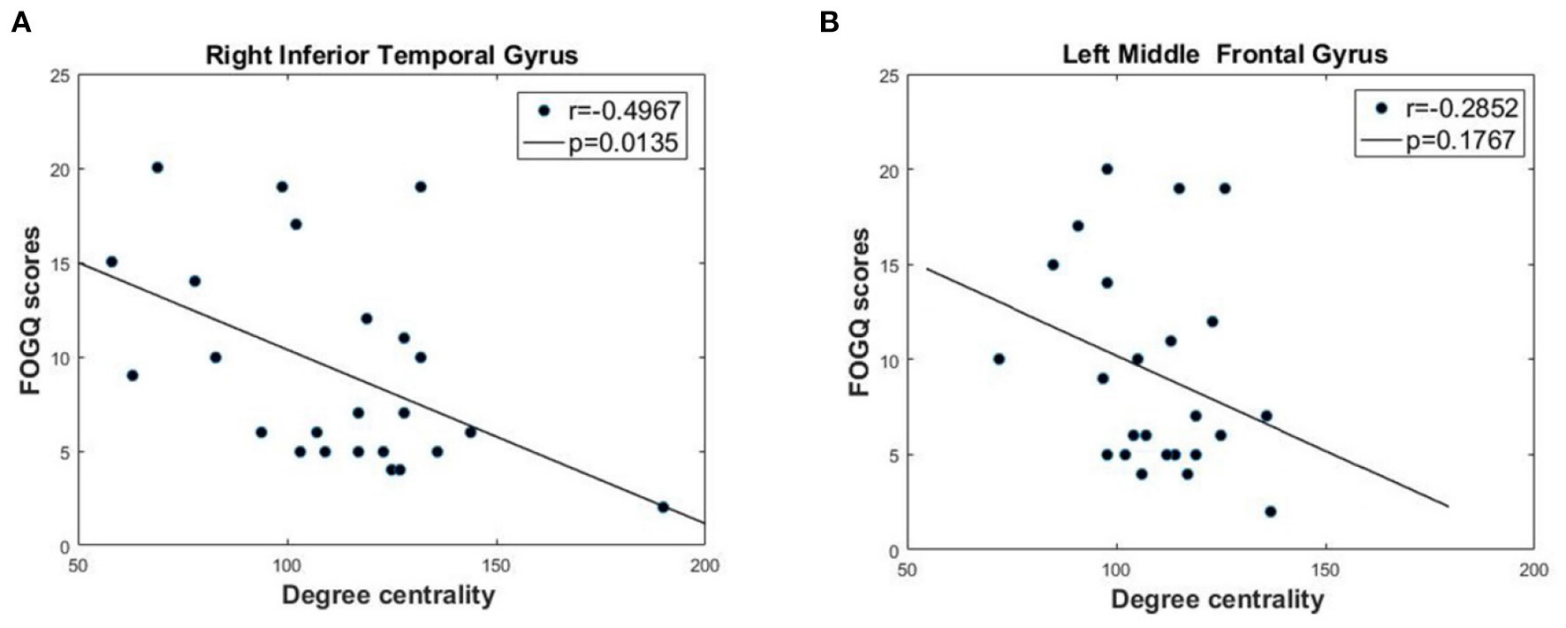
Correlation analysis between DC and FOGQ scores in right inferior temporal gyrus (ITG) and left middle frontal gyrus (MFG). **(A)** The negative correlation between DC values and FOGQ scores in right ITG. **(B)** The negative correlation between DC values and FOGQ scores in left MFG.

Based on the GTA results, we used Pearson's correlation analysis to further investigate the association between FOGQ scores and DC. As shown in Figure 3, for the two brain regions with significantly different DC in PD FOG+ vs. PD FOG–, we found that the DC in the right ITG of PD FOG+ patients was negatively correlated with their FOGQ scores (*p* = 0.0135, *r* = −0.4967) (FDR corrected). On the contrary, no significant correlation was noted between DC in the left MFG and FOGQ scores (*p* = 0.1767, *r* = −0.2852) (FDR corrected).

For the 10 brain regions with significantly different DC in PD FOG+ vs. HC, the *p*-value and *r*-value of correlation analysis is given in Table 2. Except for right ITG, DC is not significantly correlated with FOGQ score for the other nine regions (*p* > 0.05).

**Table 2:** Correlation and ROC analysis results of 10 brain regions with significantly different DC in PD FOG+ vs. HC.

**Brain regions**	**Measures of correlation analysis**		**AUC of ROC analysis**		
	***p*** **-value**	***r*** **-value**	**PD FOG+ vs. HC**	**PD FOG– vs. HC**	**PD FOG+ vs. PD FOG–**
ITG (R)	0.0135	−0.4967	0.859	0.777	0.813
MFG (L)	0.1562	−0.2763	0.682	0.673	0.536
MFG (R)	0.2574	−0.2242	0.637	0.526	0.557
STG (L)	0.1465	−0.3218	0.662	0.647	0.513
ITG (L)	0.3726	−0.1723	0.592	0.527	0.512
PhG (L)	0.2149	−0.2512	0.614	0.572	0.625
PhG (R)	0.1582	−0.3016	0.625	0.582	0.533
IPL (L)	0.3236	0.1718	0.542	0.579	0.549
IPL (R)	0.2874	0.2453	0.583	0.573	0.525
MTG (R)	0.3563	−0.1813	0.571	0.531	0.546

### Analysis of ROC Curves

**Figure 4:**
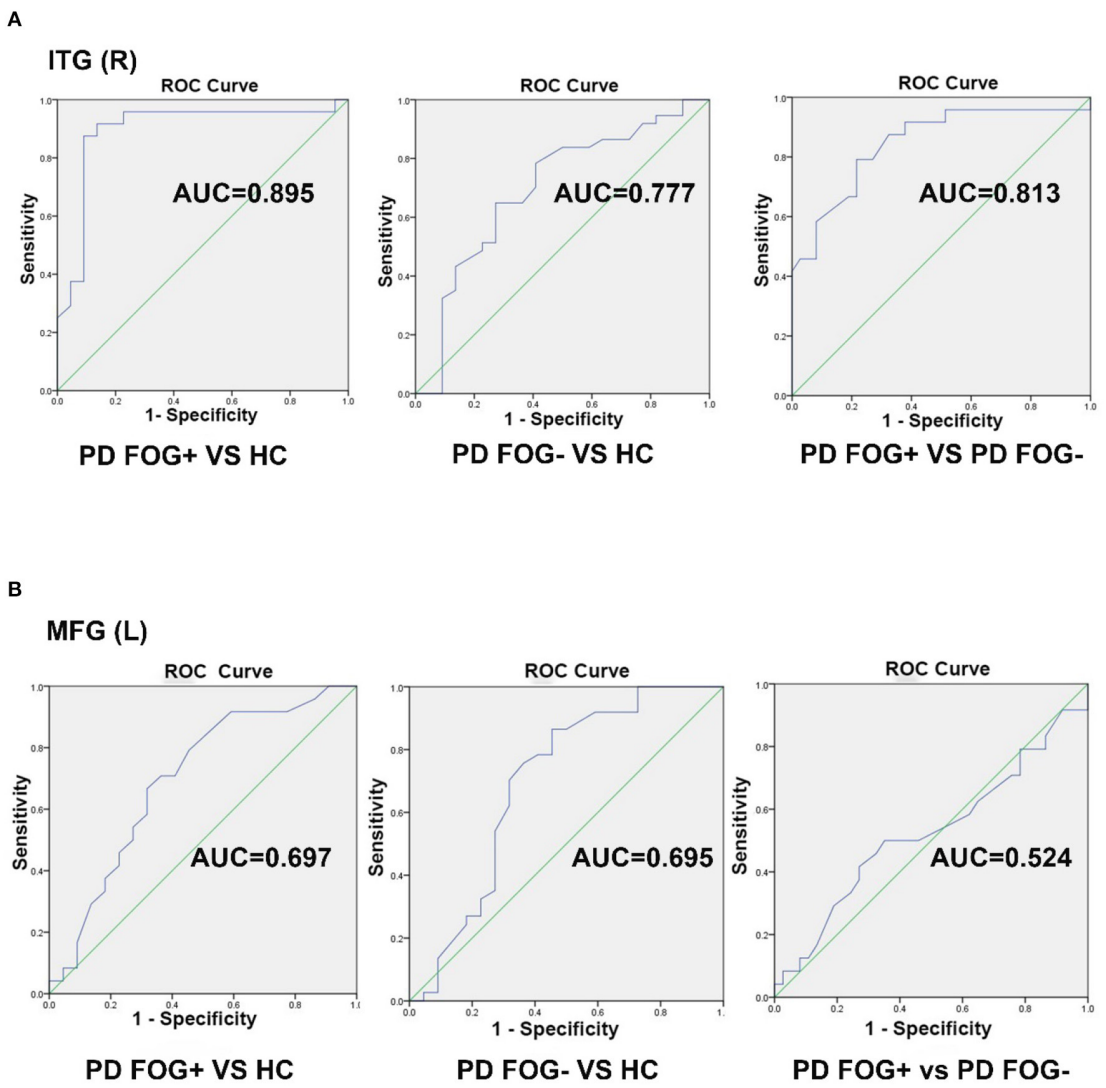
Receiver operating characteristic (ROC) curve for classifying three groups by using the DC value in the right inferior temporal gyrus (ITG) and left middle frontal gyrus (MFG). **(A)** The ROC curve of right ITG. **(B)** The ROC curve of left MFG.

In the pairwise comparison of the three study groups, we found that the DC value of the right ITG region showed a significant difference. Therefore, we set the right ITG region identified in PD FOG+ vs. PD FOG– as ROI and extracted its DC value for further ROC analysis. The ROC results of right ITG are shown in Figure 4A. The AUC of right ITG was 0.813 [95% confidence interval (CI): 0.697–0.929, *p* < 0.001] when distinguishing PD FOG+ patients from PD FOG– patients; 0.895 (95% CI: 0.788–1, *p* < 0.001) when distinguishing PD FOG+ patients from HCs; and 0.777 (95% CI: 0.641–0.913, *p* < 0.001) when distinguishing PD FOG– from HCs. Further analysis showed that the specificity for differentiating PD FOG+ from PD FOG– and HCs was relatively high, up to 91.9% (DC value of cut-off point: 119.5) and 90.9% (DC value of cut-off point: 133.0), respectively (Table 3).

**Table 3:** ROC analyses for differentiating groups.

**Brain regions**	**Categories**	**AUC**	**Cut-off point**	**Sensitivity**	**Specificity**	**p Value**	**95% CI**
ITG (R)	PD FOG+ vs. PD FOG–	0.813	119.5^a^	58.3% (14/24)	91.9% (34/37)	<0.001	0.697–0.929
	PD FOG+ vs. HC	0.895	133.0	87.5% (21/24)	90.9% (20/22)	<0.001	0.788–1.000
	PD FOG– vs. HC	0.777	151.5	76.7% (16/22)	72.7% (16/22)	<0.001	0.641–0.913
MFG (L)	PD FOG+ vs. PD FOG–	0.524	107.5	50.0% (12/24)	64.9% (25/37)	0.043	0.368–0.679
	PD FOG+ vs. HC	0.697	116.0	66.7% (16/24)	68.2% (15/22)	0.022	0.541–0.853
	PD FOG– vs. HC	0.695	122.5	86.5% (32/37)	54.5% (12/22)	0.013	0.540–0.850

*AUC, area under the curve; HC, healthy control; PD FOG+, Parkinson's disease with freezing of gait; PD FOG–, Parkinson's disease without freezing of gait; ITG, inferior temporal gyrus; MFG, middle frontal gyrus; CI, confidence interval*.

a *Using this cut-off point, the DC value of ITG could correctly identify 14 of 24 PD FOG+ patients and 34 of 37 PD FOG– patients, resulting in a sensitivity and specificity of 58.3 and 91.9%, respectively. The meanings of other cut-off points are similar*.

Similarly, we used the DC value of left MFG for further ROC analyses. As shown in Figure 4B, the AUC of left MFG was 0.524 (95% CI: 0.368–0.679) when differentiating PD FOG+ from PD FOG–; 0.697 (95% CI: 0.541–0.853) when differentiating PD FOG+ from HC; and 0.695 (95% CI: 0.540–0.850) when differentiating PD FOG– from HC. In contrast to right ITG, the DC value of left MFG did not have a good discriminative ability. Using the cut-off value of 107.5, 116.0, and 122.5, the specificity was 64.9, 68.2, and 54.5% for PD FOG+ vs. PD FOG–, PD FOG+ vs. HC, and PD FOG– vs. HC, respectively.

For the 10 brain regions with significantly different DC in PD FOG+ vs. HC, the AUC value of ROC analysis is given in Table 2. Except for right ITG, the AUC value is smaller than 0.7 for the other nine regions. It is noted that AUC of MFG (L) in PD FOG+ vs. HC (Table 2) is different from that in PD FOG+ vs. PD FOG– (Table 3). The reason is that the two subregions identified in PD FOG+ vs. HC and PD FOG+ vs. PD FOG– are different, though both belong to MFG (L). MFG (L) includes seven subregions (no. 15, 17, 19, 21, 23, 25, and 27). Our DC comparison is at the level of 246 subregions. In PD FOG+ vs. HC, the significant difference in DC is observed in the subregion of no. 15. However, in PD FOG+ vs. PD FOG–, the significant difference in DC is observed in the subregion of no. 23.

## Discussion

In this study, we constructed a functional brain network and used GTA to perform statistical analysis on DC in the PD FOG+, PD FOG–, and HC groups. The major finding of our study is that the DC among the three groups showed significant differences. Correlation analysis found that the DC of right ITG and FOGQ were negatively correlated, which indicates that the information communication ability of right ITG became poor with increasing FOG severity. The ROC results showed that the DC of the right ITG could well-distinguish PD FOG+ from the PD FOG– and HC groups. This may indicate that the right ITG region plays an important role in the physiological mechanism of PD FOG.

The ITG exhibited a lower nodal centrality in PD FOG+ patients than in PD FOG– and HCs. Nodal centrality is a powerful measure to assess the relative importance of a node in functional brain network and has been used to evaluate the information integration ability of a single region within brain networks (28). Our results showed significant differences in the information transmission and communication of the ITG region in the functional brain network.

These results are consistent with previous studies about FOG. Wang et al. found that compared with HCs and PD FOG– patients, PD FOG+ patients showed abnormal pedunculopontine nucleus functional connectivity, mainly in the visual temporal gyrus (right MTG and right ITG) (22). More recently, Jung et al. (55) showed that the connectivity between the left ITG and right fusiform gyrus is inversely proportional to FOG latency. Moreover, the study by Hu et al. demonstrated that PD FOG+ patients exhibited a higher amplitude of low frequency fluctuations (ALFF) (56) in the left ITG and lower ALFF in right MFG than both the PD FOG– and HC groups.

FOG often occurs when starting, turning, or passing through narrow aisles or doors. It takes a long time for the patient to move slowly to adapt before starting to walk again. Especially during turning, the actual movement of the two legs is different, which requires bilateral coordination and the brain to have good control over the movement of the legs. Right ITG subserves language and semantic memory processing, observation for motion, visual perception, and multimodal sensory integration. We found that decreased DC of the right ITG in PD FOG+ patients indicated the reduced ability of information integration. The decreased DC of right ITG may be interpreted as the disruption of these functions and the potential neural mechanism in PD FOG+. It has been reported that the regional homogeneity (ReHo) values of PD FOG– patients has a negative correlation with FOGQ in the left gyrus rectus and right ITG (57).

We also evaluated the predictive performance of DC in differentiating the PD FOG+ patients from PD FOG– patients and HCs. The results of ROC curve analyses indicate that DC changes in the ITG can be a potential imaging biomarker with the ability to distinguish PD FOG+ patients from PD FOG– patients or HCs. The discriminative performance has emphasized the importance of DC in ITG from another viewpoint. Further studies may integrate more discriminative features extracted from functional brain network into a model by using machine leaning methods to help precisely and objectively diagnose PD FOG+ and PD FOG– (39, 58, 59).

The behavioral domains of left MFG mainly focus on cognition, memory, and action inhibition. We did not find significant correlations between the DC value of left MFG and FOGQ scores. A significant difference was observed in the DC value of left MFG between the PD FOG+ and PD FOG– groups. The ROC results showed that the DC value of left MFG did not have a good discriminative ability.

PD FOG+ patients have more significantly different (disrupted) brain regions than PD FOG– patients; these extra brain regions may provide some explanation for the occurrence of FOG. The correlation analysis results showed a negative relationship between the DC of right ITG and FOGQ scores. This indicates that the information communication ability of ITG gets poorer with increasing severity of FOG. The different results for right ITG and left MFG may suggest that the group two-sample *t*-test is not equal to the correlation analysis, and ROC analysis. The left MFG showed significantly different DC between PD FOG+ and PD FOG–, but its DC did not significantly correlate with the FOGQ scores. Moreover, the DC of the left MFG does not present good discriminative capability among the three groups (specificity, <70.0%). Two-sample test is an intergroup study of traditional brain mapping, the correlation analysis is an intragroup study of PD FOG+, and ROC analysis is a kind of study for predictive modeling (58). Therefore, these three methods might be different and complementary. The involvement of the left MFG is not as specific to enable a good discriminative power. For the DC of the left MFG, the lack of correlation with FOGQ scores may suggest this structure is not significantly involved in FOG. Another possible reason might be that the intragroup evaluation is biased by a reduced sample size.

Our study has some limitations. First, the sample size is relatively small, which may have reduced the statistical power. Second, our study is cross-sectional; therefore, the dynamic alterations of related regions and measures cannot be examined during the progression of PD FOG+ from PD FOG–.

## Conclusion

Significant differences in nodal DC obtained from rs-fMRI-based functional brain network were observed in some brain regions in the PD FOG+, PD FOG–, and HC groups. Compared to the PD FOG– and HC groups, the PD FOG+ group showed decreased DC in the right ITG. In PD FOG+ patients, the DC of right ITG was negatively correlated with the FOGQ scores, suggesting that reduced ability of information integration may contribute to the severity of FOG. ROC analysis indicates that DC of right ITG may be a unique network biomarker for PD FOG+ and have the extraordinary ability to distinguish PD FOG+ patients from PD FOG– patients and HCs. The group two-sample *t*-test, correlation analysis, and ROC analysis are three complementary methods that can be utilized together for studies of other neurological disorders.

## Data Availability Statement

The raw data supporting the conclusions of this article will be made available by the authors, without undue reservation.

## Ethics Statement

The studies involving human participants were reviewed and approved by The Ethics Committee of Guangzhou First People's Hospital. The patients/participants provided their written informed consent to participate in this study.

## Author Contributions

CJ performed experiments and analyzed the data along with SQ. SQ, YY, and XW conceived the study, presented the results, and wrote the manuscript along with CJ. XR collected and analyzed the data. YT and CL supervised the algorithm development and analyzed the data. All authors read and approved the final manuscript.

## Funding

This work was partly supported by the National Natural Science Foundation of China under Grant (Nos. 82072008 and 81871846), the Fundamental Research Funds for the Central Universities (N181904003, N172008008, and 2124006-3), the Science and Technology Planning Project of Guangzhou (202102010020), the Natural Science Foundation of Guangdong Province (2021A1515011288), and the National Key Research and Development Plan of China (2019YFC0118805).

## Conflict of Interest

The authors declare that the research was conducted in the absence of any commercial or financial relationships that could be construed as a potential conflict of interest.

## Publisher's Note

All claims expressed in this article are solely those of the authors and do not necessarily represent those of their affiliated organizations, or those of the publisher, the editors and the reviewers. Any product that may be evaluated in this article, or claim that may be made by its manufacturer, is not guaranteed or endorsed by the publisher.

## Abbreviations

ANOVA: analysis of variance
AUC: area under the curve
BOLD: blood oxygen level dependent
CI: confidence interval
CSF: cerebrospinal fluid
DC: degree centrality
EPI: echo-planar imaging
FAB: Frontal Assessment Battery
FC: functional connectivity
FDR: false discovery rate
fMRI: functional magnetic resonance imaging
FOG: freezing of gait
FOGQ: freezing of gait questionnaire
FOV: field of view
GRETNA: GRaph thEoreTical Network Analysis
GTA: Graph theory analysis
HARS: Hamilton Anxiety Rating Scale
HC: healthy control
HDRS: Hamilton Depression Rating Scale
H&Y: Hoehn and Yahr
ICA: independent component analysis
IPL: inferior parietal lobule
ITG: inferior temporal gyrus
MFG: middle frontal gyrus
MMSE: Mini-mental State Examination
MoCA: Montreal Cognitive Assessment
MTG: middle temporal gyrus
PD: Parkinson's disease
PD FOG+: Parkinson's disease with freezing of gait
PD FOG–: Parkinson's disease without freezing of gait
PhG: parahippocampal gyrus
ROC: receiver operating characteristic
ROI: region of interest
RSFC: resting-state functional connectivity
RS-fMRI: resting state-functional magnetic resonance imaging
STG: superior temporal gyrus
TE: echo time
TR: repetition time
TUG: timed Up and Go
UPDRS: Unified Parkinson's Disease Rating Scale
WM: white matter.
